# Pharmacological and rehabilitation strategies in opioid-induced constipation: an observational study

**DOI:** 10.3389/fresc.2026.1683677

**Published:** 2026-01-21

**Authors:** Salvatore Caramma, Rosaria Condorelli, Enrico Buccheri, Marinella Maria, Giuliana Rosta, Liberato Longo, Fabrizio Li Gotti, Simona Brundo, Rita Chiaramonte

**Affiliations:** 1 Pain Management Unit, Azienda Ospedaliero Universitaria Policlinico G Rodolico San Marco, Catania, Italy; 2 Department of Medical Oncology, EOC- Repubblica e Cantone Ticino Ente Ospedaliero Cantonale, Bellinzona, Switzerland; 3 Unit of Internal Medicine, Azienda Ospedaliero Universitaria Policlinico G Rodolico San Marco, Catania, Italy; 4 Sant'Agata Rehabilitation Hospital, Catania, Italy; 5 Disability and Rehabilitation Unit, Department of Rehabilitation, Azienda Sanitaria Provinciale (ASP) of Catania, Catania, Italy

**Keywords:** analgesics, opioids, chronic pain, constipation, receptors opioid mu, rehabilitation

## Abstract

**Background:**

Research on the combined effects of Naldemedine and targeted rehabilitation for managing opioid-induced constipation (OIC) is limited. This retrospective study aims to explore their use in chronic pain patients with OIC.

**Methods:**

The study examined 53 patients with OIC, comparing outcomes between those receiving Naldemedine with targeted physical exercise (28 in Naldemedine + Rehabilitation group) and those receiving Naldemedine alone (25 in Naldemedine alone group). Assessments at baseline (T0), 14 ± 2 days (T1), and three months (T2) included Numerical Rating Scale (NRS), Patient Assessment of Constipation Quality of Life (PAC-QoL) and Bowel Function Index (BFI).

**Results:**

Combining Naldemedine with rehabilitation led to higher patient satisfaction at three months compared to Naldemedine alone, while no significant between-group differences were observed in abdominal pain relief or bowel function. After 14 days of treatment, both groups demonstrated improvements in NRS, BFI, PAC-QoL total score, and PAC-QoL subscale for satisfaction (*p* = 0.716, *p* = 0.886, *p* = 0.585, *p* = 0.431). After three-month, Naldemedine + Rehabilitation group showed significantly higher satisfaction levels compared to those on Naldemedine alone, as indicated by PAC-QoL total score and sub-score for satisfaction (*p* = 0.031, *p* = 0.0163).

**Conclusions:**

The implementation of a combined approach involving targeted physical exercise alongside Naldemedine treatment showed promising results in enhancing patients' satisfaction.

## 1 Introduction

Chronic pain is a prevalent and debilitating condition affecting millions of individuals and substantially impacting quality of life and functional capacity (1).

Opioid therapy has been reported to be associated with a reduction in pain intensity of at least 30% in both neuropathic and musculoskeletal pain conditions (2). Alongside pharmacological treatments, additional drug-based and rehabilitative strategies have been proposed for pain management (3, 4), including pain related to infection conditions (5). Nevertheless, opioids remain among the most frequently prescribed medications, despite their well-documented association with adverse events, particularly opioid-induced constipation (OIC) (6). Indeed, up to 80% of patients report at least one adverse event, with constipation (41%), nausea (32%), and somnolence (29%) being the most commonly described (2). Opioids are prescribed by a range of healthcare professionals, including internal medicine (16.4%), dentistry (15.8%), family medicine (10.3%), orthopedics (2.9%), oncology (2%), pain medicine (1.5%), and physical medicine and rehabilitation specialists (0.8%) (7). Given this broad prescribing landscape, appropriate recognition and management of OIC represent an important aspect of comprehensive patient care. Moreover, opioid therapy may be considered in selected patients with chronic non-cancer pain who have not achieved adequate symptom control with optimized non-opioid treatments (8).

Conservative approaches to OIC management, such as increased fibre and fluid intake, physical activity, and first-line therapy including standard or over-the-counter laxatives and stool softeners, are frequently insufficient. In such cases, second-line treatments may be considered, including acting μ-opioid receptor antagonists (PAMORA), such as Methylnaltrexone, Naloxegol, and Naldemedine, as well as chloride channel activators like Lubiprostone (9, 10). Naldemedine, in particular, has been reported to be well-tolerated and convenient due to its once-daily oral administration, while maintaining analgesic efficacy in patients with cancer-related and chronic non-cancer pain (11).

Despite the substantial burden of OIC on healthcare systems, and its negative association with quality of life in patients with chronic non-oncological pain (12), a considerable portion of patients remain inadequately managed. Approximately 27% report no laxative use, while an additional 25% receive insufficient treatment due to suboptimal laxative regimens (13). These findings underscore the complexity of OIC management, which may be further influenced by patient-related factors such as concerns about medication efficacy, fear of adverse effects, and perceived interference with daily activities, particularly among individuals with a less favorable attitude toward pharmacological treatments (14). Rehabilitation is recognized as a first-line non-pharmacologic intervention in chronic pain management and may offer additional benefits in this context (6). Accordingly, the integration of targeted physical exercise with pharmacological approaches to OIC warrants further investigation. To date, limited research has examined the combined use of Naldemedine and structured rehabilitation interventions in the management of OIC.

The rationale for exploring this combined approach is based on the complementary nature of the proposed interventions. Naldemedine acts peripherally on μ-opioid receptors and is intended to counteract opioid-related gastrointestinal effects. Rehabilitation strategies, such as abdominal massage, stretching, and aerobic activity, are commonly used to support bowel function through mechanical stimulation, improved abdominal wall mobility, and modulation of autonomic activity. Additionally, participation in structured exercise programs may be associated with higher levels of self-efficacy and patient engagement, factors that have been linked to improved treatment satisfaction and adherence.

From both physiological and psychological perspectives, these mechanisms provide a theoretical framework for examining whether rehabilitation may be associated with additional benefits when combined with pharmacological therapy. Indeed, from both physiological point of view, rehabilitation strategies such as abdominal massage, stretching, and light aerobic activity may enhance gastrointestinal motility and support the mechanical processes involved in defecation, potentially complementing the peripheral opioid-receptor antagonism of Naldemedine. From a psychological perspective, engaging patients in an active self-management program may increase self-efficacy, perceived control, and treatment satisfaction, which are known to positively influence adherence and overall patient-reported outcomes.

On this basis, this retrospective study aims to explore outcomes associated with the combined use of Naldemedine and targeted physical exercise in patients with musculoskeletal chronic pain experiencing OIC. A secondary objective is to assess the quality of life in relation to this combined treatment approach.

## 2 Materials and methods

Written informed consent was obtained from all participants, and the study adhered to the ethical principles of the Declaration of Helsinki. The study was approved by the Ethics Committee (Ethics Committee of Sicilia Region number 220—27/12/2024). This observational retrospective study was conducted by anaesthesiologists of Pain Management Centre, in collaboration with specialists in Physical Medicine and Rehabilitation, from September 2021 to March 2023. Following completion of data collection, the dataset underwent a period of verification, anonymization, and completeness checking. During this phase, clinical records were reviewed to ensure consistency of outcome measures and eligibility criteria. Ethical approval was subsequently obtained, after which the retrospective statistical analysis was conducted in May 2024.

The study included data from 53 patients diagnosed with OIC based on the Rome Foundation criteria (15), which considers both quantitative (less than three bowel movements per week) and qualitative symptoms (straining, hard stools, and sensation of incomplete evacuation). Inclusion criteria encompassed patients with musculoskeletal chronic pain, aged between 40 and 75 years old, previously treated with laxative without adequate improvement or satisfaction, with a Bowel Function Index (BFI) ≥ 28.8. This threshold corresponds to the validated cutoff for clinically meaningful opioid-induced constipation, as values below 28.8 are generally considered within the normal range and not indicative of clinically relevant OIC. The BFI ≥ 28.8 cutoff has been widely adopted in both clinical trials and observational studies to identify patients with significant constipation-related symptom burden attributable to opioid therapy (16). Patients were eligible for inclusion if they had completed medical records with full data available for all primary outcome measures at each assessment point (baseline, 14 ± 2 days, and three months). Patients with missing or incomplete data were excluded to ensure the reliability of the study's findings. Other exclusion criteria included oncological patients and those who had not defecated for four consecutive days, necessitating examination to exclude acute ileus. This exclusion criterion was applied to maintain a consistent and robust dataset throughout the study. Patients started this treatment due to the lack of effectiveness of conservative treatments (rehabilitation, physical therapy), NSAIDs, and infiltration therapy for pain management.

The characteristics of the study sample were summarized in Table 1.

**Table 1:** Characteristics of sample.

Groups	Numbers	Age (years)	Pain Etiology	Opioid Drugs	Drug distribution
		Sex F/M			
N + R group	28	68.07 ± 10.57	11 Low back pain;	12 Tramadol tablets;	*χ*^2^ = 0.55
		20/8	5 Polyarthralgia;	4 Oxycodone hydrochloride tablets;	
					df = 3
				9 Transdermal buprenorphine;	
			3 Fibromyalgia;	3 Transdermal fentanyl	*p* = 0.90
			9 RA		C = 0.10
N group	25	68.88 ± 10.92	10 Low back pain;	12 Tramadol tablets;	
		17/8	4 Polyarthralgia;	2 Oxycodone hydrochloride tablets;	
			3 Fibromyalgia;	8 Transdermal buprenorphine;	
			8 RA	3 Transdermal fentanyl	

N, Naldemedine; R, Rehabilitation program; N + R group, Naldemedine + Rehabilitation group; N group, Naldemedine alone group; F, females; M, males; RA, rheumatoid arthritis; *χ*^2^, chi-square test of independence; C, contingency coefficient; df, degree of freedom; *p*, *p*-value.

**Figure 1:**
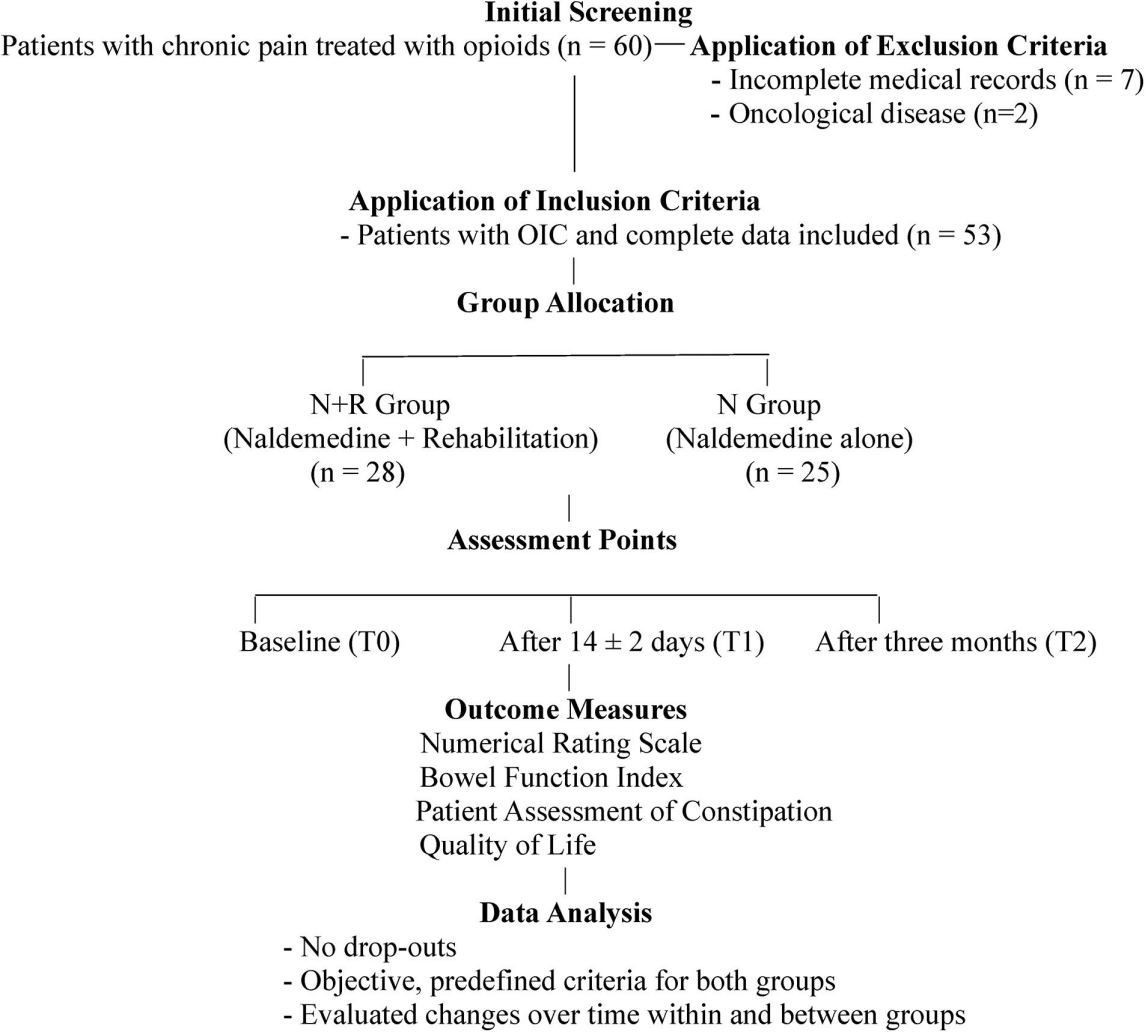
Flowchart of study design and patient selection.

A total of 60 patients with a diagnosis of opioid-induced constipation (OIC) were initially screened for eligibility. Seven patients were excluded for the following reasons: incomplete medical records preventing assessment at all required time points (*n* = 5), presence of oncological disease (*n* = 2), The remaining 53 patients met all inclusion criteria and were included in the final analysis. Participants were divided into two groups: Naldemedine + Rehabilitation group of 28 patients underwent a specific rehabilitation program and Naldemedine treatment, while 25 patients of the Naldemedine alone group declined participation rehabilitation programs, but assumed Naldemedine treatment (Figure 1). Reasons for refusal included scepticism about the additional benefits, fatigue, and discomfort due to their chronic pain.

The participants in the Naldemedine + Rehabilitation group received the rehabilitation program, which included all three exercises (abdominal massage, aerobic exercises, and abdominal muscle stretching), as indicated in Table 2. The Naldemedine alone group did not perform any rehabilitation exercises. Rehabilitation adherence in the Naldemedine + Rehabilitation group was monitored through structured follow-up evaluations at T1 and T2 using standardized interviews and patients' diaries. Patients were asked to report the frequency and regularity with which each component of the rehabilitation program was performed. These self-reports and diary entries were reviewed by the medical staff to verify consistency over time and to reinforce the correct execution of the prescribed protocol, acknowledging that adherence remained self-reported and therefore subject to potential bias.

**Table 2:** Rehabilitation program proposed for constipation in Naldemedine + Rehabilitation group.

Exercise/Training	Techniques	Position	Frequency	Adherence categories	Sources of program design
Abdominal massage	The massage followed the physiological course of the colon using clockwise movements. Techniques included stroking, effleurage, superficial and deep kneading, and final vibration of the abdominal wall.	Supine with head-of-bed elevated 30 °–45 °.	∼15 min, twice daily, after breakfast and dinner	High adherence	Yıldırım D et al. (38)
Aerobic exercises (stepping/walking)	Step-based aerobic training plus low-intensity strength/endurance exercises. (mini-squats, marching in place, hip abduction, heel raises, seated knee extension, and bridging)	Upright	∼10 min 3 times per week	High adherence	Wilson PB (40), Jensen BT et al. (41)
Abdominal muscle stretching	Three supine stretches: (a) *Wind-relieving pose*: one knee flexed toward chest, head approximated toward knee, 15–30 s each side; (b) *Knees-to-chest*: bilateral hip flexion maintained close to torso; (c) *Reclined spinal twist*: trunk and pelvis rotated with one knee flexed, held at ∼80% maximal comfortable rotation for 1 min each side.	Supine	∼5 min, 3 times per week	High adherence	Hanai A et al. (39)

High adherence ≥80% sessions.

### 2.1 Treatment, timing, assessments, and sampling strategy

Patients received continuous treatment throughout the observation period of 90 days, with a daily dose of 200 micrograms of Naldemedine in both groups, alongside self-guided rehabilitation in the Naldemedine + Rehabilitation group.

Patients were assessed at three time points: baseline (T0), before the initiation of treatment; at the first follow-up visit approximately 14 days (T1 14 ± 2) after treatment initiation; and at the subsequent three-month follow-up (T2).

In the study, three assessment scales were used to evaluate the treatment outcomes, including the Numerical Rating Scale (NRS) (17), Bowel Function Index (BFI) (18), and Patient Assessment of Constipation-Quality of Life (PAC-QoL) (19). NRS, a 10-point scale, assessed abdominal pain intensity. BFI scores, reflecting defecation difficulty, feelings of incomplete defecation, and overall defecation satisfaction over the past week, were evaluated by medical staff. The self-administered PAC-QoL was used to assess treatment outcomes and quality of life assessing physical and psychosocial discomfort worries and concerns, and satisfaction.

The diagnostic criteria for OIC in this study were based on the Rome Foundation criteria (15). Additionally, the diagnosis of chronic pain conditions such as low back pain, polyarthralgia, fibromyalgia, and rheumatoid arthritis (Table 1) was confirmed by specialists in rheumatology or relevant fields, as orthopedic, physical medicine and rehabilitation, pain management specialists. The use of validated assessment tools like BFI helped confirm the presence and severity of OIC in participants.

### 2.2 Treatment methods

All patients received oral administration of controlled-release oxycodone hydrochloride tablets (5–40 mg/day), transdermal buprenorphine (5–20 mch/h), transdermal fentanyl (12–100 mch/h), tramadol tablets (50–200 mg/day) (Table 1). Additionally, to prevent OIC, all patients were prescribed oral laxatives (15 mL of lactulose solution twice daily), and advised to maintain adequate fluid intake of at least 1.5–2 litres per day and high-fibre diet, consisting of fruits, vegetables, and whole grains, for at least the previous three months.

### 2.3 Statistical analysis

The R Statistical Software was used for data analysis. Continuous variables are presented as mean and standard deviation (SD). With the Student's *t*-test, the study compared the scores of the different assessment in the groups. For all tests, a *p*-value of less than 0.05 was considered statistically significant. Between-group effect sizes were calculated using Cohen's *d* and adjusted for small-sample bias using Hedges' *g*. Effect sizes (Hedges' g, corrected for small sample size) with 95% confidence intervals were calculated for all between-group comparisons (NRS, BFI, PAC-QoL total score, PAC-QoL satisfaction) to complement *p*-values and support interpretation of clinical relevance. No formal correction for multiple testing was applied, as all comparisons were clinically driven (NRS, BFI, PAC-QoL total and satisfaction). Given the exploratory, real-world nature of the study and the limited sample size, applying conservative corrections was considered at high risk of inflating type II error, that is, failing to detect real differences between groups despite their presence.

## 3 Results

### 3.1 Effectiveness of treatment

Of the 53 patients, 25 received Naldemedine alone, while 28 underwent rehabilitation in addition to drug therapy.

No adverse effects of Naldemedine necessitating treatment discontinuation were recorded during the study period. Some mild adverse effects, such as flatulence, were reported but were tolerable and did not require discontinuation of the treatment.

In the Naldemedine alone group, NRS scores decreased significantly from T0 to T2 (mean = 7.56–4.68), in a significative way (*p* < 0.0001). BFI scores showed a similar significant reduction from T0 to T2 (mean = 69.85–37.5) (*p* < 0.0001), and PAC-QoL total scores decreased significantly from T0 to T2 (mean = 48.12–23.24) (*p* < 0.0001). The PAC-QoL satisfaction subscale scores showed significant improvement, increasing from T0 (mean = 5.20) to T2 (mean = 12.48) (*p* < 0.0001). These findings suggest that Naldemedine therapy alone was associated with improvements in abdominal pain, symptoms, quality of life and patient satisfaction with constipation therapy.

In the Naldemedine + Rehabilitation group, NRS scores also showed a significant reduction from T0 to T2 (mean = 7.71–4.86) (*p* < 0.0001). BFI scores decreased from T0 to T2 (mean = 69.8–37.5) (*p* < 0.0001), and PAC-QoL total scores improved from T0 to T2 (mean = 48.14–26.21) (*p* < 0.0001). The PAC-QoL satisfaction subscale scores increased substantially from T0 to T2 (mean = 5.07–14.50) (*p* < 0.0001) (Table 3).

**Table 3:** Statistical results: comparison intragroup.

Pairwise comparisons N group					
Assessment	Timing	Factors	Mean difference	95% CI	*p*-value
NRS	T0	T1	1.88	1.33–2.42	**<0** **.** **0001**
	T0	T2	2.88	2.22–3.53	**<0** **.** **0001**
	T1	T2	1.00	0.58–1.42	**<0** **.** **0001**
BFI	T0	T1	−25.38	−31.50–−19.26	**<0** **.** **0001**
	T0	T2	−32.36	−38.90–−25.81	**<0** **.** **0001**
	T1	T2	−6.97	−9.12–−4.81	**<0** **.** **0001**
PAC-QoL satisfaction subscale scores	T0	T1	−4.44	−6.41–−2.46	**<0** **.** **0001**
	T0	T2	−7.28	−9.37–−5.18	**<0** **.** **0001**
	T1	T2	−2.84	−5.02–−0.65	0.0082
PAC-QoL total score	T0	T1	20.36	15.49–25.22	**<0** **.** **0001**
	T0	T2	24.88	20.14–29.61	**<0** **.** **0001**
	T1	T2	4.52	−0.92–9.96	0.1292
Pairwise comparisons Naldemedine + Rehabilitation group					
NRS	T0	T1	1.96	1.43–2.49	**<0** **.** **0001**
	T0	T2	2.85	2.32–3.39	**<0** **.** **0001**
	T1	T2	0.89	0.56–1.22	**<0** **.** **0001**
BFI	T0	T1	−24.92	−30.39–−19.46	**<0** **.** **0001**
	T0	T2	−32.33	−38.17–−26.49	**<0** **.** **0001**
	T1	T2	−7.404	−9.38–−5.42	**<0** **.** **0001**
PAC-QoL total score	T0	T1	19.00	14.25–23.74	**<0** **.** **0001**
	T0	T2	21.92	16.39–27.46	**<0** **.** **0001**
	T1	T2	2.92	−2.25–8.11	0.4827
PAC-QoL satisfaction subscale scores	T0	T1	−5.39	−7.12–−3.66	**<** **.** **0001**
	T0	T2	−9.42	−10.66–−8.19	**<0** **.** **0001**
	T1	T2	−4.03	−5.60–−2.46	**<0** **.** **0001**

N, Naldemedine; R, Rehabilitation program; N + R group, Naldemedine + Rehabilitation group; N group, Naldemedine alone group; BFI, bowel function index; NRS, numerical rating scale; PAC-QoL, patient assessment of constipation quality of life; T0, first assessment; T1, assessment after 14 days; T2, assessment after 3 months; CI, confidence interval.

Bold values indicate statistical significance.

These findings suggest that Naldemedine together with specific program of rehabilitation was associated with improvements in abdominal pain, bowel function, quality of life and patient satisfaction with constipation therapy.

### 3.2 Comparative analysis between Naldemedine alone and combined therapy

At baseline (T0), there were no significant differences between the Naldemedine-alone group and the Naldemedine + Rehabilitation group across any outcome measures, confirming sample homogeneity (all *p* > 0.05). The distribution of opioid types was also comparable between groups at baseline (*χ*² = 0.555, df = 3, *p* = 0.906), indicating no statistically significant imbalance in opioid treatment characteristics between the Naldemedine alone group and Naldemedine + Rehabilitation groups (Table 1). At T1, both groups showed significant within-group improvements in all measures, with no statistically significant between-group differences. By T2, between-group comparisons revealed statistically significant differences only for patient-reported satisfaction outcomes. Specifically, the Naldemedine + Rehabilitation group showed greater improvement in PAC-QoL total scores (*p* < 0.0001) and in the PAC-QoL satisfaction subscale (*p* = 0.016), compared with Naldemedine alone (Table 4).

**Table 4:** Statistical results: intergroups comparison.

Score	N group			N + R group			*t*-test			Effect sizes between-groups at T2		
	T0	T1	T2	T0	T1	T2	T0	T1	T2	Hedges' *g*	95% CI	Interpretation
NRS	7.56 ± 1.12	5.68 ± 0.69	4.68 ± 0.63	7.71 ± 1.24	5.75 ± 0.70	4.86 ± 0.70	*p* = 0.63	*p* = 0.716	*p* = 0.340	0.27	−0.28–0.81	Small effect
BFI	69.85 ± 9.53	44.5 ± 12.4	37.5 ± 13.40	69.8 ± 9.01	44.9 ± 11.8	37.5 ± 12.6	*p* = 0.99	*p* = 0.886	*p* = 0.990	0.00	−0.54–0.54	No effect
PAC-QoL	48.12 ± 8.69	27.76 ± 8.4	23.24 ± 8.20	48.14 ± 8.57	29.14 ± 9.75	26.21 ± 11.2	*p* = 0.99	*p* = 0.585	*p* < 0.0001	0.30	–0.23–0.84	Small effect
Sub Pac-QoL	5.2 ± 1.98	9.64 ± 4.01	12.48 ± 3.68	5.07 ± 1.90	10.46 ± 3.56	14.5 ± 1.60	*p* = 0.84	*p* = 0.431	*p* = 0.016	0.72	0.16–1.27	Moderate effect

N, Naldemedine; R, Rehabilitation program; N + R group, Naldemedine + Rehabilitation group; N group, Naldemedine alone group; BFI, bowel function index; NRS, numerical rating scale; PAC-QoL, patient assessment of constipation quality of life; T0, first assessment; T1, assessment after 14 days; T2, assessment after 3 months; PAC-QoL, satisfaction scale scores; SubPac-QoL, satisfaction subscale scores; *p*, *p*-value; 95% CI, 95% confidence intervals.

To quantify the magnitude of these differences, effect sizes were calculated using Cohen's *d* and corrected with Hedges' *g*. At T2, between-group effect sizes were small for NRS (*g* = 0.27; 95% CI −0.28–0.81), BFI (*g* = 0.00; 95% CI −0.54–0.54), and PAC-QoL total score (*g* = 0.30; 95% CI −0.25–0.84), indicating minimal clinical divergence between groups for pain and bowel-function outcomes. In contrast, the PAC-QoL satisfaction subscale demonstrated a moderate effect size (Hedges' *g* = 0.72; 95% CI 0.16–1.27), consistent with the observed statistically significant difference and suggesting a clinically meaningful advantage of the combined Naldemedine-plus-rehabilitation approach specifically in patient satisfaction. Notably, the improvement in satisfaction occurred despite comparable changes in abdominal pain and bowel-function measures. Indeed, it indicated that enhanced satisfaction was independent of physiological bowel outcomes and may reflect experiential or patient-centered benefits rather than improved motility.

## 4 Discussion

The rationale for this study arose from the high rate of inadequate response to laxatives among patients with OIC (14). This clinical challenge highlights the need for structured treatment approaches for OIC in patients with chronic musculoskeletal pain. In this retrospective study, data were collected from patients with OIC treated with Naldemedine, either alone or in combination with a targeted physical exercise program. Outcomes related to on abdominal pain, bowel function and quality of life were examined and compared between the two treatment approaches.

Over time, improvements in abdominal pain, bowel function, and constipation-related quality of life were observed in both groups. However, the combined Naldemedine + rehabilitation approach was not associated with greater improvements in abdominal pain or bowel function compared with Naldemedine alone. The only consistent between-group difference was observed in patient satisfaction at three months, which was higher in the combined treatment group. This finding suggests a supportive rather than synergistic contribution of rehabilitation. The dissociation between stable bowel-function outcomes and increased satisfaction may indicate that the observed benefit of rehabilitation was primarily related to psychosocial and behavioural factors rather than to enhanced intestinal motility (20). Participation in a structured program may be associated with greater self-efficacy, engagement in symptom management, and perceived control over the condition, all of which have been linked to higher patient-reported satisfaction and well-being (21). Accordingly, the present findings do not support an additive physiological effect of rehabilitation on bowel function. Instead, they suggest a patient-centred benefit reflected in higher satisfaction. Given the observational design and the use of self-selected groups, these between-group differences should be interpreted with caution. Group allocation was based on patients' willingness to participate in the rehabilitation program. The retrospective and non-randomized nature of the study precludes any causal inference.

Different opioid agents are known to vary in their association with constipation. For example, tapentadol and some opioid-naloxone combinations have been reported to be associated with lower rates of constipation compared with traditional µ-agonists (22). Dose reduction or opioid rotation may also be considered when opioids must be continued (23). Although baseline demographic and clinical characteristics were generally balanced between groups, the distribution of specific opioid agents differed. This imbalance could influence bowel-function outcomes and should be considered a potential confounding factor.

The study population included patients with heterogeneous musculoskeletal pain conditions. Differences in pain etiology may have influenced baseline functional status, response to rehabilitation, and the relationship between pain, activity level, and bowel function. The effects of rehabilitation on mobility, abdominal muscle control, and symptom perception may vary across conditions, such as axial low-back pain vs. osteoarthritis. Consequently, the generalizability of these findings to specific diagnostic subgroup is limited. Future studies may benefit from focusing on more homogeneous cohorts or from conducting stratified analyses across pain diagnoses.

Current standards for constipation management, including OIC, includes lifestyle interventions such as increased fibre intake, adequate hydration, and physical activity, along with laxatives (e.g., osmotic, stimulant, or stool softeners) as first-line therapy. In patients with an inadequate response, opioid receptor antagonists such as Naldemedine, Naloxegol, and Methylnaltrexone are considered appropriate options. These agents target the gastrointestinal effects of opioids without interfering with analgesia and are commonly used when conventional laxative therapy is insufficient. This study contributes to the ongoing discussion on how to manage unavoidable opioid-related side effects and underscores the importance of integrated pharmacological and non-pharmacological strategies.

Early intensification of prophylactic laxative treatment, especially during opioid doses escalation, has been suggested as a strategy to limit the development of OIC (24). Peripherally PAMORAS have been shown to be associated with sustained improvements in bowel function and quality of life (25, 26). Among these agents, Naldemedine has been reported as one of the most effective options (27) and, together with naloxone-containing formulations, appears to have a favorable safety profile (28).

According to the CDC Clinical Practice Guideline (6), non-opioid therapies are preferred for managing subacute and chronic pain conditions such as back pain, fibromyalgia, and hip or knee osteoarthritis.

In line with these recommendations, an Italian multidisciplinary panel (29) proposed a comprehensive strategy for the prevention and management of OIC, including conservative interventions aimed at improving fibre intake, hydration, and mobility while maintaining pain control (29).

When conservative measures are insufficient, osmotic or stimulant laxatives are recommended as first-line pharmacological therapy, with combination regimens used in cases of partial response (29). PAMORAs are suggested as second-line treatment in patients with inadequate response to laxatives, while secretagogues such as lubiprostone are reserved for selected cases and specialist settings (30).

Evidence from oncological populations suggests that rehabilitation-based interventions, including exercise, osteopathy, and acupuncture, may be associated with improvements in constipation symptoms and pain (31). In adults with chronic non-cancer pain, treatment with Naldemedine has also been associated with improvements in constipation-related outcomes (32). However, rehabilitation interventions appear more consistently linked to subjective outcomes such as satisfaction and perceived well-being than to objective bowel measures. Programs are known to enhance self-efficacy, related to patients' confidence in their ability to manage symptoms and adhere to therapeutic behaviors, which has been consistently associated with improved subjective outcomes and satisfaction in chronic conditions (33). Higher self-efficacy has been shown to correlate with greater engagement in exercise-based and self-management interventions and with improved perceived health status also independent of measurable physiological improvements (34). Indeed, the role of rehabilitation is complementary and supportive in constipation management (10). For example, abdominal massage has been shown to alleviate constipation-related symptoms without reducing laxative use, supporting its role as a complementary rather than substitutive intervention (35). Structured rehabilitation may also enhance patient engagement and perceived involvement in care, which are recognized determinant of patient satisfaction and perceived treatment success. Qualitative studies in OIC and other chronic conditions indicate that satisfaction is strongly influenced by perceived control, reassurance, education and active participation rather than symptom frequency alone (1, 36). These mechanisms may explain the higher satisfaction observed in the combined treatment group despite comparable bowel-function outcomes.

First, rehabilitation interventions (abdominal massage, light aerobic activity, stretching) frequently produce symptomatic relief—reduced bloating, less abdominal discomfort, and improved bodily awareness—that may not fully translate into changes in quantitative indices such as BFI, patient perception and well-being. Evidence suggests that abdominal massage and exercise can reduce constipation symptoms and improve quality of life even where their role is still unclear and objective metrics show modest change (35, 37). Moreover, participation in a structured program enhances self-efficacy and perceived control—factors consistently associated with improved patient-reported outcomes across chronic conditions—which likely contributed to higher satisfaction in the Naldemedine + Rehabilitation group. Thus, the observed pattern (subjective benefit > objective change) plausibly reflects symptomatic relief and psychosocial mechanisms in addition to direct alterations in gut motility. Hence, a program combining rehabilitation and PAMORA strategies could potentially enhance outcomes without increasing the dosage and the pharmacological side effects of PAMORAs. These observations support the need for shared strategy aimed at partial symptom control and improved satisfaction, recognizing that even modest improvements in constipation can be meaningful for patients (14, 29).

Self-guided rehabilitation strategies have been described as feasible approaches for constipation management. Abdominal massage has been associated with modest increases in defecation freqeuency by 13% and improvements in constipation symptoms (38). Stretching of the abdominal musculature and education on defecation posture are also commonly recommended (14, 39). Regular physical activity, adapted to pain and fatigue levels, is considered relevant, as low physical activity has been associated with constipation in several populations (40, 41).

Taken together, the findings suggest that combining pharmacological and rehabilitative strategies was associated with improvements in OIC-related outcomes and higher patient satisfaction. The higher satisfaction reported in the combined treatment group likely reflects enhanced self-efficacy, symptom awareness, and perceived control rather than superior physiological effects as bowel-function outcomes. Previous research has shown that patients may report better health status and satisfaction when they feel more capable of managing their condition, even when objective clinical changes are limited (42). Subjective improvements such as reduced bloating, abdominal discomfort, and muscle tension have been reported with abdominal massage (43) and rehabilitation programs (12) and may contribute to perceived treatment effectiveness, independently of measurable changes in bowel function.

The retrospective design of this study is associated with limitations inherent to observational research, including potential information and selection biases. In addition, the study included a heterogeneous patient population with diverse underlying conditions and treatment histories, which could complicate data analysis and interpretation. Several planned comparisons were performed, which may increase the risk of Type I error (the possibility of identifying a difference that is actually due to chance). No correction for multiple testing was applied, as the analyses were clinically driven, and the small sample size could have increased the risk of Type II error (failing to detect a real effect) applying conservative adjustments. For this reason, the findings—particularly those related to secondary outcomes—should be interpreted with caution. Between-group comparisons at each time point were conducted using independent t-tests. This approach results as an additional limitation, because it does not account for the repeated-measures structure of the data. Mixed-effects models were not applied because of the limited sample size and the exploratory nature of the study, which could have affected model stability. Group allocation was not randomized. Participation in the rehabilitation program depended solely on patient willingness, introducing potential self-selection bias. Patients who opted to participate may have differed in motivated, health awareness, or attitudes toward active self-management. These factors may have influenced patient-reported satisfaction independently of the intervention and limit the interpretation of between-group differences. This limitation must be considered when interpreting the findings and limits the ability to draw causal inferences. Adherence to rehabilitation program was assessed through patient self-report supported by exercise diaries, without objective monitoring or direct supervision. While this approach reflects routine clinical practice, it may be subject to reporting bias and may not fully capture actual adherence. The absence of objective adherence measures limits the precision with which the contribution of the rehabilitation program to patient-reported outcomes, particularly satisfaction, can be interpreted. Although no statistically significant differences in opioid type distribution were observed between groups, residual confounding cannot be excluded. Opioid agents differ in their association with constipation. The small sample size with limited representation of specific opioid categories may have reduced the ability to detect subtle imbalances. This factor should be considered when interpreting bowel-function outcomes. Residual confounding by opioid type therefore cannot be fully excluded and should be addressed in future studies with larger, prospectively balanced cohorts.

## 5 Conclusion

This real-world, retrospective study suggests that both groups treated with Naldemedine alone and those receiving Naldemedine combined with rehabilitation experienced significant improvements over time in pain, bowel function, and constipation-related quality of life. Between-group differences were statistically significant only for patient satisfaction at 3 months, not for bowel function or pain outcomes. No additional benefits of rehabilitation were detected for abdominal pain or objective bowel function outcomes. These findings suggest that rehabilitation may provide a supportive, experiential and synergistic benefit, potentially enhancing patients' perceived satisfaction with treatment, rather than a clear physiological advantage on constipation-related measures. Given the observational design and self-selection into treatment groups, these results are preliminary and do not establish causality. Randomized controlled trials are warranted to determine whether rehabilitation offers a true additive benefit and to better define its role within multimodal management strategies for opioid-induced constipation.

## Data Availability

The raw data supporting the conclusions of this article will be made available by the authors, without undue reservation.
